# The Effect of a Surgical Skills Course on Confidence Levels of Rural General Practitioners: An Observational Study

**DOI:** 10.1055/s-0036-1593737

**Published:** 2016-10-25

**Authors:** Pippa Byrd, Olga Ward, Jeffrey Hamdorf

**Affiliations:** 1 Clinical Training and Evaluation Centre, School of Surgery, The University of Western Australia, Crawley, Western Australia, Australia

**Keywords:** surgical procedures, family physician, training, teaching, rural health

## Abstract

**Objective** To investigate the effect of a short surgical skills course on general practitioners' confidence levels to perform procedural skills.

**Design** Prospective observational study.

**Setting** The Clinical Evaluation and Training Centre, a practical skills-based educational facility, at The University of Western Australia.

**Participants** Medical practitioners who participated in these courses. Nurses, physiotherapists, and medical students were excluded. The response rate was 61% with 61 participants providing 788 responses for pre- and postcourse confidence levels regarding various surgical skills.

**Intervention** One- to two-day surgical skills courses consisting of presentations, demonstrations, and practical stations, facilitated by specialists.

**Main Outcome Measures** A two-page precourse and postcourse questionnaire was administered to medical practitioners on the day. Participants rated their confidence levels to perform skills addressed during the course on a 4-point Likert scale.

**Results** Of the 788 responses regarding confidence levels, 621 were rated as improved postcourse, 163 were rated as no change, and 4 were rated as lower postcourse. Seven of the courses showed a 25% median increase in confidence levels, and one course demonstrated a 50% median increase. All courses showed statistically significant results ( *p* < 0.001).

**Conclusion** A short surgical skills course resulted in a statistically significant improvement in the confidence levels of rural general practitioners to perform these skills.

Australian rural general practitioners (GPs) encounter a diverse range of presentations including those that require procedural intervention. Competent and confident GPs are a valuable resource, particularly in Western Australia, where access to both routine and emergency procedures is challenging because of the degree of geographical isolation. As such, it is important that rural GPs are adequately trained to perform certain surgical procedures and have the confidence to carry out these procedures independently.

When rural GPs want to increase or maintain their surgical skills, they face several challenges, including financial and time constraints and difficulty finding locum relief. 1

Local evidence suggests that where rural hospitals are accredited for procedural care, the quality of that care provided is on par with care expected in urban hospitals. 2 If rural GPs can improve their confidence levels through further training, it is likely that improved job satisfaction and greater retention of health professionals in rural areas will result. This will result in improved access to locally performed procedures for rural patients.

The current literature shows only a small amount of evidence of varying quality relating to this topic. Previous studies have measured baseline levels of confidence and the effect of courses on GPs' confidence levels. 3 4 Other studies monitored performance rates or competency of surgical skills following an intervention. 5 6 7

## Methods

This study was performed to test the hypothesis that rural GPs who participated in a short surgical skills training course would have an improvement in confidence levels to perform these skills.

This prospective observational study exposed participants to surgical skills courses from the Cutting Edge series conducted at The Clinical Training and Evaluation Centre (CTEC) at The University of Western Australia. CTEC is a practical skills-based educational facility that recreates an authentic clinical environment for learning. The content of CTEC courses have been informed by training needs analysis to identify gaps between the current level of skills of medical professionals and the skills required to deliver safe and effective health care. 8 9 Data were drawn from the courses offered at CTEC during 2014, which included seven different courses, with one being offered twice, for a total of eight courses.

The topics addressed in each course, for which presentations, demonstrations, and practical stations are provided, are detailed in Table 1 . Courses utilize fresh frozen cadaveric models, prosections, simulation models, and human models to assist with learning practical skills. 8

**Table 1:** Topics addressed in surgical skills courses

Course	Topics addressed
Essential Surgical Skills	• Instrumentation, knots, and local anesthesia • Suturing technique • Lesion biopsy • Wound management
Managing Skin and Soft Tissue Injuries	• Wounds, open fractures, and foreign bodies (dirty and delayed) • Lipomas and cysts • Skin biopsy • Minor burns • Keloid scarring
Peripheral Nerve Block Course	• Faciomaxillary and jaw • Paravertebral and intercostal • Lower limb and femoral/sciatic • Upper limb and brachial plexus
Procedural Obstetrics and Gynaecological Skills	• Ectopic pregnancy • Perineal and anal sphincter repairs • Hysteroscopy and endometrial biopsy • Postpartum hemorrhage • Enterotomy and cystotomy • Bartholin cysts • Cesarean sections
Advanced Procedures	• Tendon release and carpal tunnel release • Vasectomy • Burr holes • Fasciotomy • Nail procedures and podiatric surgery
Advanced Skin Procedures	• Planning excisions and safe margins • Wedge resections • Flaps • Skin grafts
Emergency Procedures Practical Course	• Airway/surgical airway • Ear, nose, and throat, ophthalmology, and dental • Hemostasis and burns • Trauma and chest • Neurosurgical emergencies • Obstetric emergencies • Urological emergencies

CTEC courses are delivered by procedural specialists and are limited to small groups of between 10 and 20 participants. Participants receive access to online resources to allow for essential prereading. The group is divided to rotate through stations lasting 30 to 45 minutes each. This time allows for adequate practice and gives specialist instructors the opportunity to provide individual feedback. Some courses include a discussion of case-based scenarios to reinforce learning.

External referencing is sought for The Cutting Edge courses through accreditation by The Royal Australian College of General Practitioners. Four of the seven courses are also accredited with The Australian College of Rural and Remote Medicine, and the remaining three courses have applications pending.

The data were collected from doctors who participated in this course. Nurse practitioners, physiotherapists, and medical students also participated or observed but were excluded from the analysis. Participants were not actively recruited for the purpose of research. Consent was provided by participants, and ethics approval was not required for this study.

Data were collected from two-page pre- and postcourse questionnaires completed on the day of the course. Participants rated their confidence on a 4-point Likert scale. The collected data were analyzed using IBM SPSS version 23 (Armonk, New York). All courses were analyzed independently due to variation in the intervention. Because the data were ordinal, a Wilcoxon signed rank test was used to determine whether changes in confidence levels postcourse, in comparison to precourse, were statistically significant. 10 A *p* value of <0.05 was considered statistically significant. It was not appropriate to analyze the data from course 8 with a Wilcoxon signed rank test due to the asymmetrical distribution of the differences between the two groups. 10 Therefore, a sign test was used to analyze the data from this course. 10

## Results

One hundred participants registered to complete eight Cutting Edge Series courses during 2014, and 61 participants had answers included in the analysis of this study. Of the total participants, 36 identified their vocation as GPs, 13 as GP proceduralists, 5 as junior doctors, and 7 did not specify their seniority. The majority of participants were currently practicing in Western Australia (50), with some from interstate (6 in South Australia, 2 in Victoria, 1 in Queensland). Twenty-nine participants identified rural locations as their only place of practice, and 21 participants also practiced in metropolitan areas.

Of the 788 items rated by 61 participants, improvements in postcourse confidence levels were shown in 621 of the items (79%). One hundred sixty-three items were rated as having no improvement, and four items that were rated lower in confidence following the surgical skills course.

All courses demonstrated a statistically significant increase in confidence levels of 1 to 2 points or 25 to 50%. Seven of these courses showed a median increase in confidence levels of 25% ( *p* < 0.001), with one course showing a median increase of 50% in confidence levels ( *p* < 0.001).

## Discussion

Several characteristics of this study lend validity to the results obtained. A prospective observational study design was the most appropriate design. The effect of confounding factors has been reduced by administering questionnaires on the day of the course, eliminating the impact of further learning or clinical experience over time. The use of training needs analysis ensured relevant skills were chosen for the course, so results could translate to clinic practice. Other factors, such as small group numbers, a high demonstrator-to-participant ratio, prereading, opportunity for practice, and the opportunity for feedback, also increased the quality and effectiveness of this intervention.

The surrounding evidence currently available in this field, although not providing a direct comparison, provides context to our results. Tolhurst et al conducted a study in 1999, which focused on measuring the baseline levels of confidence of rural GPs to perform certain procedures. 3 Although this information is slightly outdated, it gives us valuable information regarding rural GPs in Australia. With a large sample size of 84 and a reliable 6-point Likert scale as measurement, this study noted that over half of the GPs on call for hospital emergency cases rated their confidence as 3 or less with certain procedural skills. 3 Detailed background information regarding the sample group also provided more meaningful results.

A 2006 study by Lopez et al evaluated GP confidence levels following participation in the Early Management of Severe Trauma course. 4 This was a well-designed study, with 122 GPs completing a two-page survey at various times following the course (up to 15 years). In this study, there was no measurement of baseline levels of confidence to give an accurate representation of the effect of the course itself. This study did not consider exposure to additional training courses or frequency of skill performance. Results demonstrated a statistically significant improvement in GP confidence levels. Although this study did not intervene with training specifically for surgical skills, it emphasizes the positive impact training can have on GP confidence levels.

Another aspect, which proves relevant when discussing this topic, is the measurement of performance rates of surgical procedures following the intervention of a skills course. Two studies suggest that performance rates correlate closely with GPs' confidence levels. Maguire in 2000 studied a 3-month intervention (meetings, surgical clinics, and a log diary). 5 In this study, selection bias is a concern with a small sample size of five and the loss of follow-up of three of the potential eight respondents. 5 Variability in the exposure during the intervention and the risk of recording bias could also affect the validity of the results. 5 Taking these issues into account, this study showed twice the amount of procedures being performed in the follow-up period and a decrease in referral of minor surgical cases from 22 to 1.4%. 5 A second, similar, study by Gmajnić et al in 2008 was conducted in rural Croatia. 6 This study followed 59 GPs who participated in theoretical education and practical work on models. 6 Although Croatia is geographically very distant from Western Australia, it appears that the procedures typically performed by GPs are similar. This study had an adequate sample size and provided good information regarding baseline characteristics. This study also found that performance rates nearly doubled, although the applicability to the Western Australia health system could still be questioned.

A final study, conducted by Jansen et al in the Netherlands in 2000, measured both performance rates and competency in technical clinical skills. 7 This study was a randomized controlled trial with an adequate sample size of 59. 7 This study, however, was subject to recall and reporting bias by relying on a diary to measure performance rates. 7 The measurement for competency levels appeared more comprehensive with 15 multiple-choice questions used to assess each skill. 7 Competency was found to significantly improve based on knowledge regarding these skills, although increased performance rates were found for only some of the skills addressed. 7

Despite the quality of our study, the methodology could have been adjusted to reduce the potential sources of bias. The Likert scale was a simple format and appropriate way to measure confidence levels, yet a scale of greater than 4 points might have offered increased sensitivity and specificity. There was no long-term follow-up to provide information about the longevity of the results. Participants were not excluded from the analysis if they did not entirely complete both questionnaires. It is uncertain how missing answers could affect the validity of results obtained.

Greater detail regarding the participants' baseline characteristics (years of experience, other qualifications, and classification of remoteness of their practice) could have provided more context for the results. The participant group was not actively recruited for the purpose of research and therefore may not accurately represent the cohort of rural GPs in Western Australia. Those practitioners who have significant barriers to participation, such as increased remoteness of location, baseline high levels of confidence to perform surgical skills, or disinterest in further education or training, would not necessarily be represented.

Of the 788 responses received, four items were rated lower in confidence following the surgical skills course. Most likely this is due to an increase in knowledge regarding correct procedure or technique and participants re-evaluating their previous confidence levels. This may encourage participants to work within their scope of practice or seek further training if required, improving the safe delivery of health care services.

Due to time limitations of this course, competency of baseline skill levels and follow-up skill levels have not been measured during this study. Although this would be useful information to have available, it provides a basis for further work to be completed in this area. If a large randomized control trial were to focus on an objective measurement, the validity of the results found in this study could be tested. Previous work has been conducted to evaluate the most effective way to objectively monitor competency of performing surgical skills. A 2013 article by Gallagher et al discussed the available evidence that evaluates the advantages and disadvantages of hand movement tracking, eye movement tracking, and direct and thorough observation by procedure experts. 11 They have suggested a combination of methods, along with tracking of errors, would be the optimal way to measure competency. 11 This information could be applied to our local population to find optimal training methods. To address this issue, GPs are provided with a certificate of participation upon completion of the course. If they are expanding their inpatient surgical procedure range, they must demonstrate their new skills under supervision to satisfy a hospital credentialing committee of their increased competence.

The results found from our study demonstrate a positive impact of a short surgical skills course and may result in improved support for GPs to access ongoing training of this kind. These results may also encourage other practitioners to be more inclined to pursue ongoing training courses to increase their confidence to practice these skills and increase the safety and quality of health care delivered in rural WA.
